# Supplementary material for: Oxidative Stress Plays an Important Role in Glutamatergic Excitotoxicity-Induced Cochlear Synaptopathy: Implication for Therapeutic Molecules Screening
Source: Antioxidants (Basel). 2024 Jan 25;13(2):149. doi: 10.3390/antiox13020149 (PMC10886292; doi:10.3390/antiox13020149)
Supplement: Supplementary file 1 [file antioxidants-13-00149-s001.zip › antioxidants-2795770-supplementary.pdf]

**Supplementary file**

**Supplementary Figure 1**

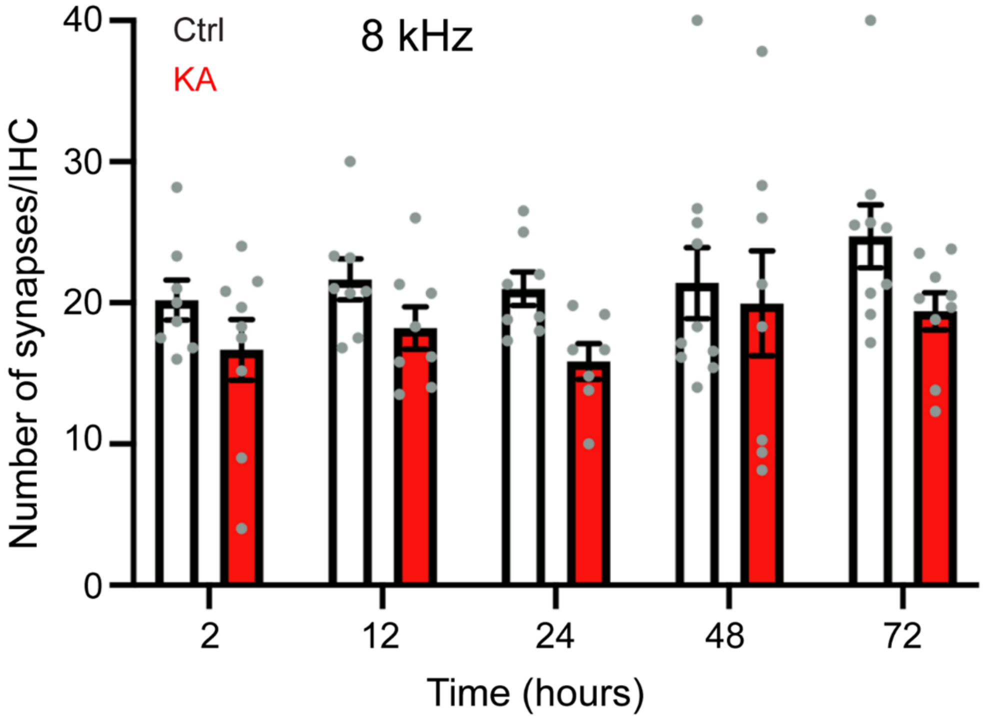


**Figure S1: Kainate did not affect IHC synapse at cochlear apical region**

Counting of the IHC synapses (paired CtBP2-PSD95) over time in cochlear region coding 8 KHz in control condition (black) and KA condition (red). All data are expressed as mean ± SEM (n = 6-10 cochleae per condition). One way ANOVA test was followed by Dunn's test.

**Supplementary Figure 2**

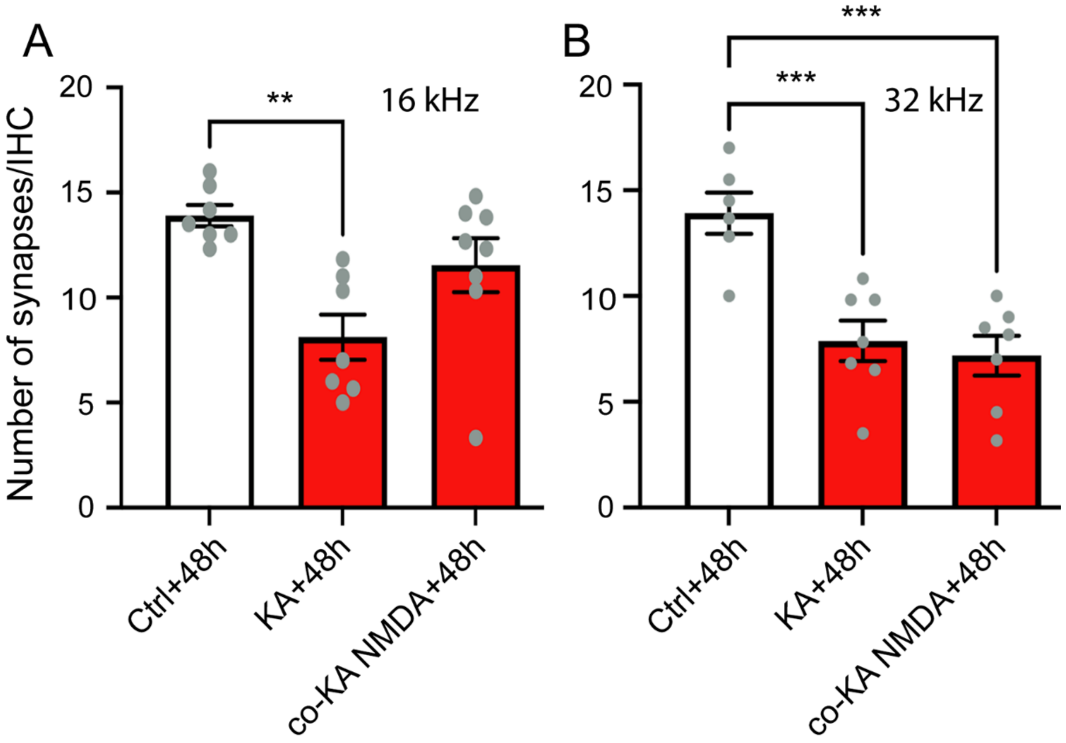


**Figure S2: Addition of NMDA did not modify the effect of kainite on IHC synapses**

**A-B**: Quantification of synapses (paired CtBP2-PSD95) per IHC in cochlear regions coding 16 KHz (**A**) and 32 KHz (**B**) of explants exposed to medium alone for 2 h + 48 h (Ctrl + 48 h), medium containing KA or KA in combination with NMDA at 0.5mM respectively for 2 h then medium alone for 48 h (KA + 48 h) or (co-KA NMDA+48h). All data are expressed as mean ± SEM (n = 6-7 cochleae per condition). One way ANOVA test was followed by Dunn's test: **p⩽0.01, ***p⩽0.001.

## Supplementary Table 1

| | target name | Antibody | Species | Isotype | Cat Number | Company | Dilution |
|---|---|---|---|---|---|---|---|
| IF Primary antibodies | V-GLUT 3 | Anti-VGLUT3 | guinea pig | IgG | 135204 RRID:AB_2619825 | Synaptic Systems | 1/500 |
| | Neurofilament 200 | Anti-NF200 | rabitt | IgG | N0142 RRID:AB_477257 | Sigma-Aldrich | 1/400 |
| | Cytochrome c oxidase | Anti-cytochrome c oxidase | mouse | IgG | 556432 RRID:AB_396416 | BD Biosciences | 1/200 |
| | BDNF | anti-BDNF | rabitt | IgG | ab223354 | Abcam | 1/500 |
| | TrkB | anti-TrkB | rabitt | IgG | ab18987 RRID:AB_444716 | Abcam | 1/500 |
| | CtBP2(ribbon) | anti-CtBP2 | mouse | IgG1 | 612044 RRID:AB_399431 | BD Biosciences | 1/500 |
| | PSD95(post synapytic density) | anti-PSD95 | mouse | IgG2a | 75-028 RRID:AB_2292909 | NeuroMab | 1/500 |
| IF Secondary Antibodies & Fluorescent | | anti-guinea pig ,Alexa 488 | goat | IgG | A-11073 RRID: AB_2534117 | Molecular Probes | 1/1000 |
| | | anti-mouse, Alexa 488 | donkey | IgG | A-21202 RRID: AB-141607 | Molecular Probes | 1/1000 |
| | | anti-rabbit,Alexa 594 | donkey | IgG | A-21207, RRID:AB_141637 | Molecular Probes | 1/1000 |
| | | anti-mouse IgG1,Alexa 488 | goat | IgG | A21121 RRID:AB_2535764 | Molecular Probes | 1/1000 |
| | | anti-mouse IgG2a, Alexa 568 | goat | IgG | A21134 RRID:AB_2535773 | Molecular Probes | 1/1000 |
| | | anti-guinea pig, Alexa 647 | goat | IgG | A21450 RRID:AB_2535867 | Molecular Probes | 1/1000 |
| | Hoechst 33342 | | | | #62249 | Thermo Fisher Scientific | 1/5000 |
| | Actin - Alexa 647 phalloidin | | | | #A22287 | Thermo Fisher Scientific | 1/1000 |

**Table S1. Shown are the antibodies used and basic information about these antibodies.**

**Supplementary Table 2**

| | Track1 | Track2 | Track3 | Track4 |
|---|---|---|---|---|
| | Chanel1 | Chanel2 | Chanel3 | Chanel4 |
| Contrast method | fluorescence | fluorescence | fluorescence | fluorescence |
| Pinhole | 0,99 AU | 1 AU | 0,99 AU | 1 AU |
| Laser wavelength | 405nm:0,4% | 488: 2,40% | 561nm: 2,40% | 633nm:1,80% |
| Scan mode | Frame | Frame | Frame | Frame |
| Scan zoom | 2,2 | 2,2 | 2,2 | 2,2 |
| Pixel time | 1,02 µs | 1,02 µs | 1,02 µs | 1,02 µs |
| Line time | 0,24 µs | 0,24 µs | 0,24 µs | 0,24 µs |
| Frame time | 2,90 s | 2,90 s | 2,90 s | 2,90 s |
| Scan direction | unidirectional | unidirectional | unidirectional | unidirectional |
| Averaging | 2 | 2 | 2 | 2 |
| Speed | 7 | 7 | 7 | 7 |
| Chanel name | Ch2GaAsP-T1 | Ch2GaAsP-T2 | Ch2GaAsP-T3 | Ch2GaAsP-T3 |
| Chanel color | Blue | Green | Red | Magenta |
| Excitation wavelength | 405 | 488 | 561 | 633 |
| Emission wavelength | 453 | 519 | 600 | 707 |
| Effective NA | 1,4 | 1,4 | 1,4 | 1,4 |
| Detection wavelength | 415-490 | 498-541 | 570-630 | 660-754 |
| Detector gain | 500 | 500 | 500 | 540 |
| Detector offset | 0 | 0 | 0 | 0 |
| Detector digital gain | 1 | 1 | 1 | 1 |

**Table S2. Shown are the parameters used for confocal image acquisitions.**
